# Direct energy spectrum measurement of X‐ray from a clinical linac

**DOI:** 10.1002/acm2.13354

**Published:** 2021-07-16

**Authors:** Yuhi Suda, Masatsugu Hariu, Ryohei Yamauchi, Ryohei Miyasaka, Atsushi Myojoyama, Weishan Chang, Hidetoshi Saitoh

**Affiliations:** ^1^ Graduate School of Human Health Sciences Tokyo Metropolitan University Tokyo Japan; ^2^ Department of Radiotherapy Tokyo Metropolitan Cancer and Infectious Diseases Center Komagome Hospital Tokyo Japan; ^3^ Department of Radiation Oncology International Medical Center Saitama Medical University Saitama Japan; ^4^ Department of Radiation Oncology St. Luke’s International Hospital Tokyo Japan; ^5^ Department of Radiation Oncology Chiba Cancer Center Chiba Japan

**Keywords:** direct measurement, energy spectrum, linac, NaI(Tl) scintillation detector, ultralow dose rate

## Abstract

A realistic X‐ray energy spectrum is essential for accurate dose calculation using the Monte Carlo (MC) algorithm. An energy spectrum for dose calculation in the radiation treatment planning system is modeled using the MC algorithm and adjusted to obtain acceptable agreement with the measured percent depth dose (PDD) and off‐axis ratio. The simulated energy spectrum may not consistently reproduce a realistic energy spectrum. Therefore, direct measurement of the X‐ray energy spectrum from a linac is necessary to obtain a realistic spectrum. Previous studies have measured low photon fluence directly, but the measurement was performed with a nonclinical linac with a thick target and a long target‐to‐detector distance. In this study, an X‐ray energy spectrum from a clinical linac was directly measured using a NaI(Tl) scintillator at an ultralow dose rate achieved by adjusting the gun grid voltage. The measured energy spectrum was unfolded by the Gold algorithm and compared with a simulated spectrum using statistical tests. Furthermore, the PDD was calculated using an unfolded energy spectrum and a simulated energy spectrum was compared with the measured PDD to evaluate the validity of the unfolded energy spectrum. Consequently, there was no significant difference between the unfolded and simulated energy spectra by nonparametric, Wilcoxon's rank‐sum, chi‐square, and two‐sample Kolmogorov–Smirnov tests with a significance level of 0.05. However, the PDD calculated from the unfolded energy spectrum better agreed with the measured compared to the calculated PDD results from the simulated energy spectrum. The adjustment of the incident electron parameters using MC simulation is sensitive and takes time. Therefore, it is desirable to obtain the energy spectrum by direct measurement. Thus, a method to obtain the realistic energy spectrum by direct measurement was proposed in this study.

## 1 INTRODUCTION

The Monte Carlo (MC) algorithm is becoming the mainstream dose calculation method in modern radiation treatment planning systems. A realistic X‐ray energy spectrum is essential for accurate dose computations using the MC algorithm. It is modeled with the MC simulation and adjusted to match the measured percent depth dose (PDD) and off‐axis ratio (OAR). However, the simulated energy spectrum may not always reproduce a realistic energy spectrum because of the physical model, simulation parameters, and simplification of the treatment head.^1^ Therefore, direct measurement of the X‐ray energy spectrum from a linac is necessary for accurate dose calculation.

Direct measurement of X‐ray energy spectrum from a clinical linac is challenging because of the high photon fluence.^2^, ^3^, ^4^, ^5^ As a result, several indirect techniques, such as Compton spectroscopy^6^, ^7^, ^8^ and transmission measurements^2^, ^3^, ^9^, ^10^, ^11^, ^12^ were performed to obtain the energy spectrum. In Compton spectroscopy, the energy spectrum of the Compton scattered photons was measured at an arbitrary scatter angle, and the energy spectrum of the primary photons was reconstructed using the Klein–Nishina formula. Levy et al.^6^, ^7^ and Landry et al.^8^ performed Compton spectroscopy for an old‐type linac using a NaI(Tl) or Ge(Li) detector. A thin carbon or aluminum plate scatterer was set at the beam axis to allow Compton scattering. The detector was shielded with lead thicker than 20 cm, and it had a pinhole to allow only Compton scattered photons to pass. For Compton spectroscopy, an incredibly massive lead is required to shield the leakage from the linac head and scattered photons from the wall.^5^ Setting accuracy is also important because the reconstructed energy spectrum depends on the scattering angle. In addition, there is a trade‐off between photon fluence and energy resolution when deciding the scattering angle.

The transmission method has been studied since the early 1980s.^2^, ^3^, ^9^, ^10^, ^11^, ^12^ In this method, transmission through an attenuator was measured using an ionization chamber. An X‐ray energy spectrum was reconstructed using the attenuation coefficient and energy response of the chamber. For the megavoltage X‐ray, the gradual change in the attenuation coefficient as a function of photon energy leads to difficulty in reconstructing the energy spectrum.^2^, ^13^ Ali et al.^9^, ^10^, ^11^ resolved this problem by measurement using attenuators and buildup caps made of several materials. The responses of several attenuators and buildup caps at each energy were required, and the extracameral effect, for example, the signal caused by scattered photons into the cable, cannot be ignored for the transmission method.^9^ In addition to the Compton spectroscopy and transmission measurements, some studies attempted to direct measurements under low photon fluence rates performed using a nonclinical linac with a thick target and a long target‐to‐detector distance.^1^, ^14^

In this study, an X‐ray energy spectrum from a clinical linac was directly measured at an ultralow dose rate to realize photon flux less than or equal to one photon pulse^−1^ at the surface of a NaI(Tl) scintillator. The measured energy spectrum was compared with an MC‐modeled energy spectrum to validate the direct measurement method. Furthermore, the PDDs based on both energy spectra were compared with the measured PDDs using an ionization chamber.

## 2 MATERIALS AND METHODS

### 2.1 Adjusting dose rate for direct energy spectroscopy

An X‐ray from a linac is generated as a pulse of a few microseconds duration and a few milliseconds interval. For a typical clinical linac (Clinac 21EX, Varian Medical System), the pulse repetition frequency is 180 s^−1^ at a dose rate of 3 Gy min^−1^ and the photon fluence rate is approximately 6.6 × 10^7^ photon cm^−2^ pulse^−1^ for 6 MV. The fluence rate of the primary photon ${\dot{\Phi }}_{\text{prim}}$ should be less than or equal to one photon pulse^−1^ to avoid a pileup in the direct measurement of the X‐ray energy spectrum using a scintillation detector. The absorbed dose rate $\dot{D}$ (Gy s^−1^) for direct measurement can be calculated using the following equation:

(1) $$\dot{D}={\dot{\Phi }}_{\text{prim}}\;f_{p}\int_{0}^{hv_{\max}}\mathrm{hv}\;p(hv)\;\left(\frac{\mu_{\mathrm{en}}(hv)}{\rho }\right)dhv,$$

where *f*_p_ is the pulse repetition frequency (pulse s^−1^), *hv* is the photon energy, *p*(*hv*) is the ratio of photon fluence at *hv* to the total fluence of the primary photon, and *µ*_en_(*hv*)/*ρ* is the mass‐energy absorption coefficient (cm^2^ g^−1^) at *hv*. According to Equation (1), the dose rate should be reduced to 3.1 nGy s^−1^ (187 nGy min^−1^) at 180 pulses s^−1^ for a 6 MV X‐ray and source to isocenter = 100 cm. Various techniques have been explored to decrease the dose rate, including (a) reducing the filament current of the electron gun, (b) adjusting the voltage on the gun grid, and (c) deliberately determine the phase difference between the electron gun and klystron.^15^, ^16^ For example, Okamoto et al.^17^ reported the linear X‐ray energy from a Clinac 21 EX at an ultralow dose rate. The gun grid voltage adjusting technique was adopted in this report since the ultralow dose rate can be achieved with similar equipment.

The dose rate was measured using an ionization chamber survey meter (ICS‐323C, Hitachi Aloka Medical) with a large ionization volume of 400 cm^3^ for adjusting the gun gird voltage so that the dose rate would be 180 nGy min^−1^. The survey meter was located at the isocenter and a polymethyl methacrylate slab of 1.5 cm thickness was placed in front of the chamber to establish buildup. The reason for using a survey meter was that the change of accumulated charge could not be obtained even with 1 h measurements at an ultralow dose rate when using a Farmer‐type chamber.

### 2.2 Verification of energy spectral invariance at a low dose rate

The tissue–phantom ratio in water at depths of 20 and 10 g cm^−2^ for a field size of 10 × 10 cm^2^, *TPR*_20,10_ was measured and determined as illustrated below to confirm the consistency of the energy spectrum between normal and ultralow dose rates^18^

(2) $$TPR_{20,10}=\frac{M(d=20\;g\;{\text{cm}}^{‐2},A=10\times 10\;{\text{cm}}^{2})}{M(d=10\;g\;{\text{cm}}^{‐2},A=10\times 10\;{\text{cm}}^{2})},$$

where *M* is the electrometer readings at the isocenter, *d* is the depth, and *A* is the field size. *TPR*_20,10_ was measured using a Farmer‐type chamber (TM30013, PTW) connected to an electrometer (UNIDOS webline, PTW). As mentioned above, since accumulated charge could not be obtained at an ultralow dose rate when using the Farmer‐type chamber, the dose rate was adjusted to 170 µGy min^−1^ for *TPR*_20,10_ measurements. The standard deviation of the five measurements was within 1% at the lowest range of the electrometer. The *TPR*_20,10_ was compared at normal and low dose rates of 3 Gy min^−1^ and 170 µGy min^−1^, respectively.

### 2.3 Direct energy spectroscopy using a NaI(Tl) scintillation detector

The energy spectrum was measured using a 7.6 cm diameter (ϕ) × 7.6 cm thickness cylindrical NaI(Tl) scintillation detector with a photomultiplier (76B76/3M, SCIONIX) connected to a multichannel analyzer (MCA) (digiBASE, ORTEC). The maximum energy was assumed to be 8 MeV, and the channel was calibrated using ^137^Cs (0.662 MeV) and ^60^Co (1.173, 1.332 MeV) calibration sources.

**FIGURE 1:**
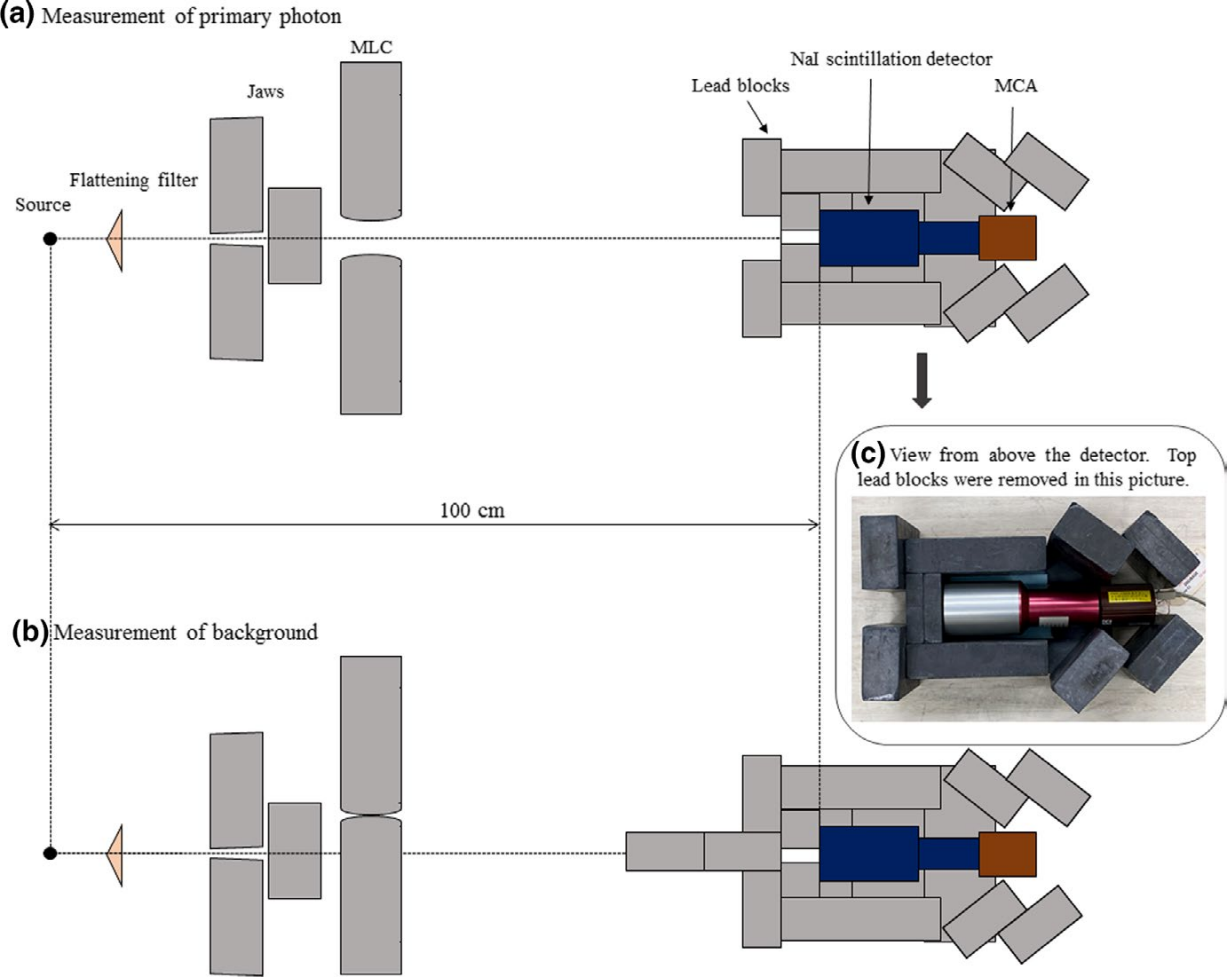
Geometry of X‐ray energy spectral measurement

The geometry for the direct measurement of the energy spectrum is presented in Figure 1. The gantry angle was set to 90°, and the distance between the source and surface of the detector was 100 cm. Several lead blocks of 5 × 10 × 20 cm^3^ were placed around the detector to shield against leakage from the linac head and scattered photons from the wall. The lead block with a 0.5 cmϕ hole was arranged in front of the detector to pass primary photons, and the radiation field was set to 0.5 × 0.5 cm^2^ with the jaws and multileaf collimator (MLC). When the dose rate was 180 nGy min^−1^, the number of primary photons that reach the detector would be close to one photon per pulse in geometry (a). However, the leakage and scattered photons could impinge on the detector even though the detector was shielded with 152 kg of lead blocks close to the couch's tolerance load. Therefore, the leakage and scattered photon counts *c*_b_ were measured with the primary photons and shielded by the MLC and lead blocks of 20 cm thickness, as shown in geometry (b). The primary photon count *c* was then obtained by subtracting count *c*_b_ from count *c*_a_ in geometry (a) as follows:

(3) $$c=c_{a}‐c_{b}.$$

The energy spectrum was measured several times for 20 min each and analyzed using the MCA emulation program (Spectrum Navigator, SEIKO EG&G). The actual energy width was 8 keV per channel, but the sum of 25 channels was used to count the energy width of 0.2 MeV to reduce statistical uncertainty.

### 2.4 Unfolding the measured spectrum

The unfolding of the measured energy spectrum is necessary to correct the Compton continuum. The Gold algorithm^19^ was adopted because it is reliable with respect to deconvolution.^20^ After *m* + 1 iterations, the count of *i*‐th channel $n_{i}^{m+1}$ can be deconvolved using the measured count of channel *i*‐th *c_i_*, response function matrix of the detector *a_ij_*, count of channel *i*‐th $n_{i}^{m}$, and count of channel *j*‐th $n_{j}^{m}$ at *m* iterations:

(4) $$n_{i}^{m+1}=\frac{n_{i}^{m}\;\;c_{i}}{\sum_{j=1}^{n}a_{\mathrm{ij}}\;n_{j}^{m}}.$$

In this equation, the response function of the NaI(Tl) scintillation detector is necessary to unfold the measured data. There are two methods to determine the response function: MC simulation and measurement using radioisotopes.^21^ In this study, the response function was obtained by MC simulation using an EGSnrc code system^22^ since no radioisotope emits gamma‐ray with energy higher than 5.0 MeV. A 7.6 cmϕ × 7.6 cm thickness cylindrical NaI crystal was reconstructed in the MC simulation code. A monoenergetic parallel photon beam of 0.5 cmϕ impinged on the center of the crystal. The energy deposition to NaI for each history was simulated and counts for each bin with an energy width of 0.2 MeV were accumulated. Table 1 lists an overview of the simulation according to AAPM TG‐268.^23^

**TABLE 1:** Overview of the MC simulation of the response function, energy spectrum, and PDD

Item name	Simulation		
	Response function	Energy spectrum	PDD
Code, version	EGSnrc,^22^ 2018	BEAMnrc,^24^ 2013	DOSXYZnrc,^25^ 2018
Geometry	Geometry macros: $CYLNDR and $PLAN2P	‐	Voxels: 1 × 1 × 0.5 cm^3^ for energy spectrum simulation 0.1 × 0.1 × 0.3 cm^3^ for PDD comparison
Materials and cross section	PEGS4	PEGS4	PEGS4
Source	Monoenergetic photons: 0.1–7.9 MeV	Electron: 6.1 MeV with 3% energy distribution	Photons according to measured energy spectrum
Transport parameters	ECUT = 0.7 MeV PCUT = 0.1 MeV Energy bin width = 0.2 MeV	ECUT = 0.7 MeV PCUT = 0.1 MeV Energy bin width = 0.2 MeV	ECUT = 0.521 MeV PCUT = 0.01 MeV
Histories	5 × 10^5^	3 × 10^9^	4 × 10^9^
Scored quantities	Energy deposition in the NaI crystal	Energy and number of photons	Energy deposition in voxels
Postprocessing	Results are not filtered	Results are not filtered	Results are not filtered

The simulated response function could not consider the energy resolution, which is the spread of the photopeak distribution of a NaI(Tl) detector. The energy resolution *R* is defined as the full width at half‐maximum (FWHM) of a particular photopeak divided by incident photon energy, and that is inversely proportional to the square root of incident photon energy.^26^ *R* can be estimated using the following equation by constant value *K* and incident photon energy *E* ^27^:

(5) $$R=\frac{K}{\sqrt{E}}.$$

Since *R* can be estimated by obtaining the *K*, FWHM of the photopeak distribution can be calculated for each incident photon energy value. *K* was derived by measurements using radioisotopes of ^137^Cs (0.662 MeV) and ^60^Co (1.173, 1.332 MeV). In the measurement, the source to detector distance was 100 cm, and the detector was shielded by lead blocks with a 0.5 cmϕ hole as in the measurement of the response function. Since the photopeak distribution forms a Gaussian curve, calculated FWHM was used to predict the spread of the photopeak by fitting it to a Gaussian distribution.^28^ The calculated spread of the photopeak distribution was compared with the measured value and incorporated into the calculated response function.

Also, calculated photopeak efficiency *ε*_p_ was compared with measured one to verify the validity of the calculation. Photopeak efficiency can be estimated using the following equation:

(6) $$\varepsilon_{p}=\frac{C_{p}}{C_{I}},$$

where *C*_p_ and *C*_I_ are the photopeak and incident photon count, respectively. The calculation parameter of *ε*_p_ in EGSnrc was the same as the calculation of response function, and the geometry of measurement of *ε*_p_ was the same as the measurement of energy resolution. The measured *C*_p_ was calculated from the peak area subtracted by the background area, which is the area below the linear line at the peak edge.^29^ Calculated *ε*_p_ was compared with measured one using radioisotopes of ^57^Co (0.122 MeV), ^137^Cs (0.662 MeV), and ^60^Co (1.173, 1.332 MeV).

### 2.5 Energy spectrum by MC simulation

The photon energy spectrum from the linac was simulated to compare the measured energy spectrum. The linac head model was reproduced on the BEAMnrc code using the geometrical and material information provided by the manufacturer.^24^ Field size was set to 10 × 10 cm^2^ and the energy spectrum was sampled. PDD and OAR were calculated using the simulated energy spectrum by the DOSXYZnrc code.^25^ The energy and spatial distribution of the incident electron were adjusted so that the simulated and measured dose distribution in the water agreed within 1%. The overview of the MC simulation is shown in Table 1.

### 2.6 Evaluation of the measured energy spectrum

The measured energy spectrum was compared with the simulated spectrum. The simulated energy spectrum was sampled from the phase space file at a region of 3 × 3 cm^2^ near the beam axis. Furthermore, the PDD for the 3 × 3 cm^2^ field was calculated by DOSXYZnrc code^25^ using the measured energy spectrum and simulated energy spectrum and compared with the measured PDD. The history of the incident particles was decided such that the simulation uncertainty was less than 0.5%. The PDD measurement was performed using a pinpoint ionization chamber (TM31016, PTW) and water phantom (MP3‐M, PTW). Agreement between the calculated and measured PDDs was estimated using relative root mean square deviation (rRMSD) as follows:

(7) $$rRMSD=\sqrt{\frac{1}{n}\;\sum_{i=1}^{n}\;{\left(\frac{PDD_{i}^{\text{cal}}‐PDD_{i}^{\text{meas}}}{PDD_{i}^{\text{meas}}}\right)}^{2}},$$

where $PDD_{i}^{\text{cal}}$ and $PDD_{i}^{\text{meas}}$ are the calculated and measured PDDs at depth *i*, respectively.

## 3 RESULTS

### 3.1 Verification of the energy spectral invariance at a low dose rate

Table 2 lists *TPR*_20,10_ at normal and low dose rates. *TPR*_20,10_ was 0.665 at a normal condition and 0.667 ± 0.004 at a low dose rate. As a result, it was confirmed that the difference in energy spectra between the normal and low dose rates was negligible.

**TABLE 2:** *TPR*_20,10_ at normal and low dose rates

	Normal	Low
Dose rate	3 Gy min^−1^	170 µGy min^−1^
*TPR*_20,10_ ± SD	0.665	0.667 ± 0.004

### 3.2 Response function

**FIGURE 2:**
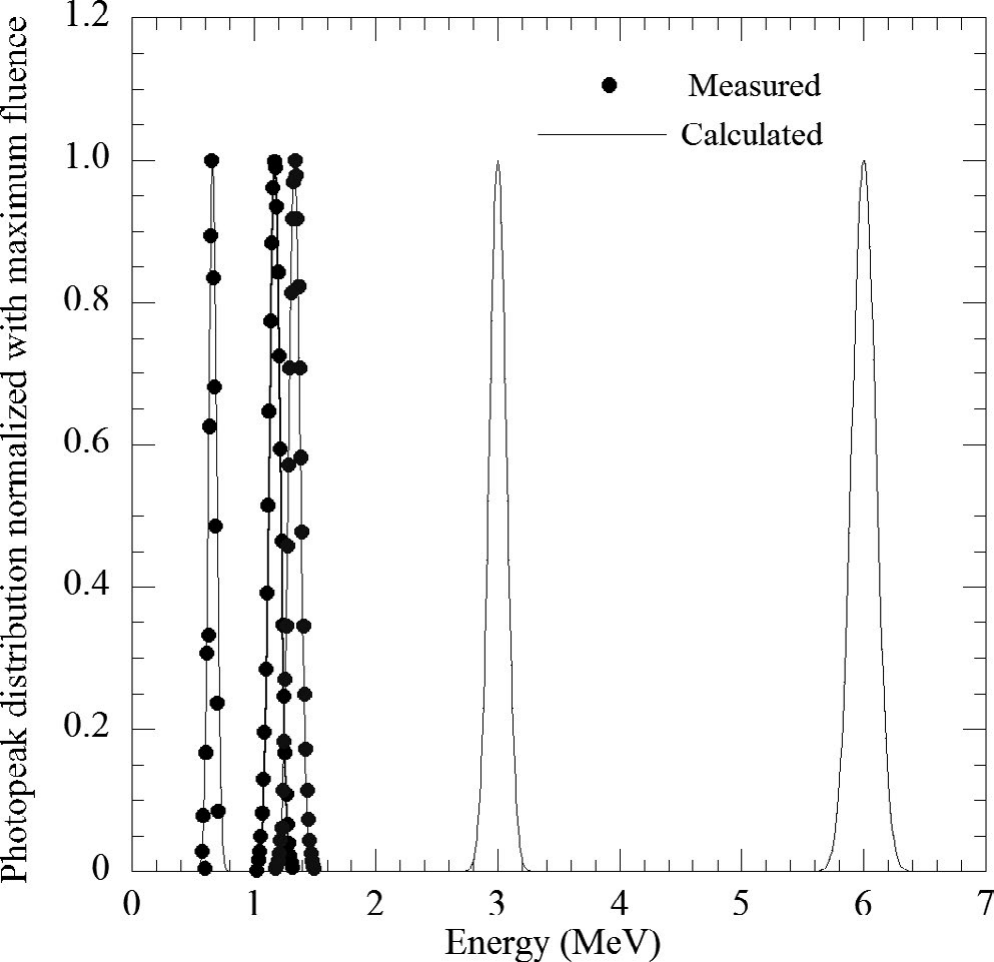
Comparison of relative photopeak distribution between measurement and calculation. Measurement was performed using ^137^Cs (0.662 MeV) and ^60^Cs (1.173, 1.332 MeV) and calculation was estimated by Equation (5) and Gaussian fitting

Figure 2 displays the comparison of relative photopeak distributions between calculation and measurement. The maximum fluence of all photopeak distributions is normalized to 1. The spread of the photopeak distribution increases with energy. According to the measured results, constant *K* in Equation (5) was derived as 9.53, and the spread of photopeak distribution is estimated using Equation (5) and Gaussian fitting. For 0.662, 1.173, and 1.332 MeV, the calculated photopeak distributions agreed with the measured distributions.

**FIGURE 3:**
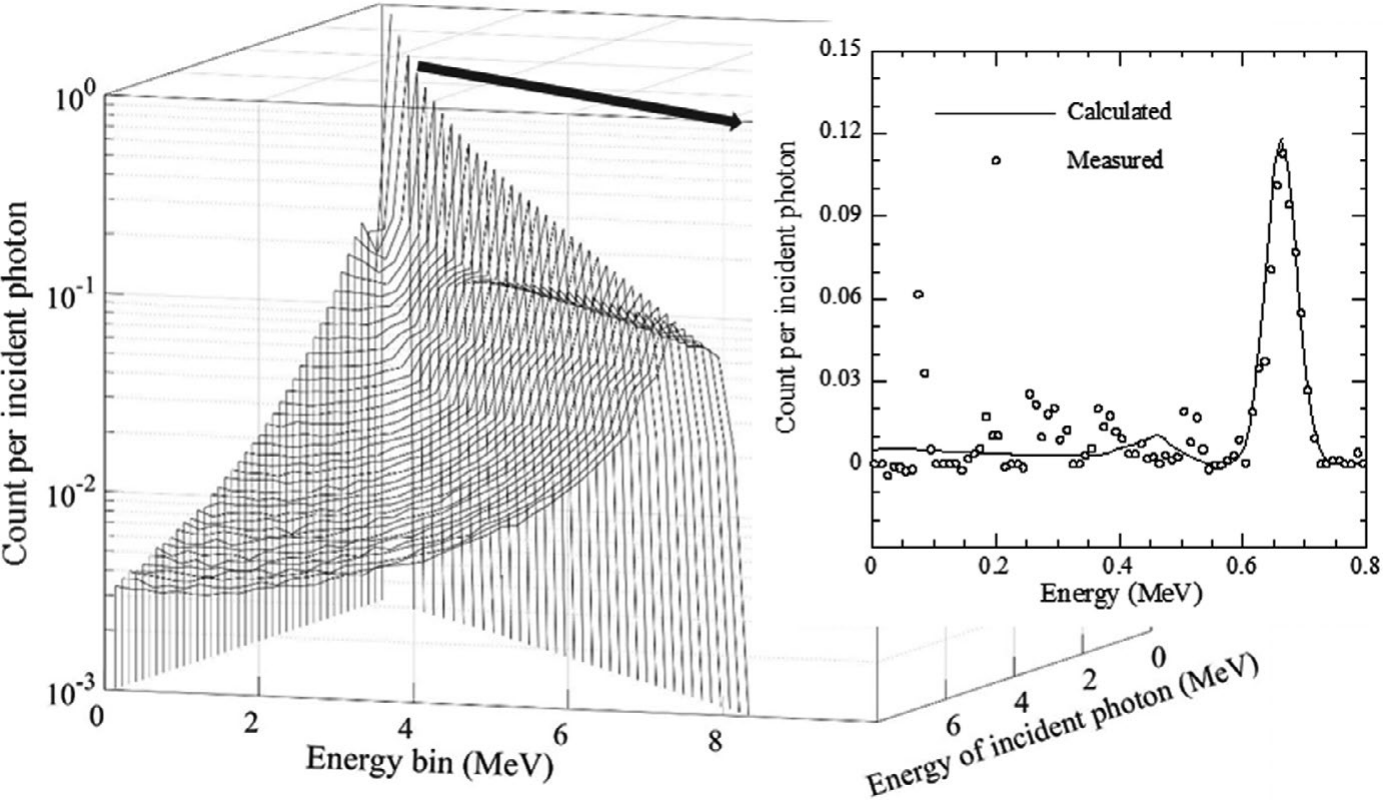
Calculated response function for a 0.5 cmϕ parallel beam and comparison of count per incident photon with measured at 0.662 MeV (^137^Cs)

Figure 3 shows the calculated response function and comparison with the measured response at 0.662 MeV (^137^Cs γ‐ray). The distribution of the photopeak widened, and total efficiency decreased as the energy increased. In the comparison at 0.662 MeV, the measured energy spectrum was noisy in the section of the Compton continuum, but the two distributions had a similar trend except the first peak seems to be noise or characteristic X‐rays of lead.

**FIGURE 4:**
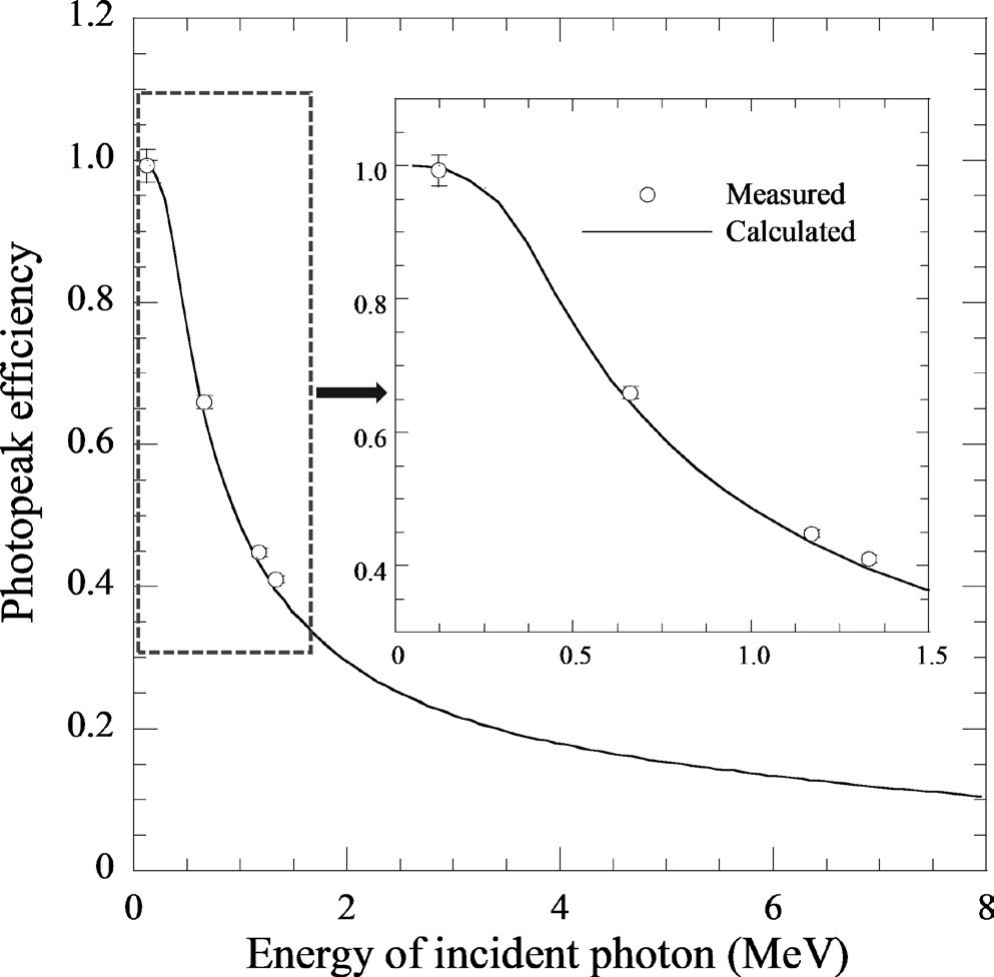
Comparison of photopeak efficiency between calculation (for 0.5 cmϕ parallel beam) and measurement. Measurement was performed using ^57^Co (0.122 MeV), ^137^Cs (0.662 MeV), and ^60^Cs (1.173, 1.332 MeV)

A comparison of the *ε*_p_ between the calculation and measurement using radioisotopes of ^57^Co (0.122 MeV), ^137^Cs (0.662 MeV), and ^60^Cs (1.173, 1.332 MeV) is displayed in Figure 4. The photopeak efficiency decreased as the incident energy increased, and the calculated *ε*_p_ agreed with the measured value within 3%.

### 3.3 Comparison of energy spectra

**FIGURE 5:**
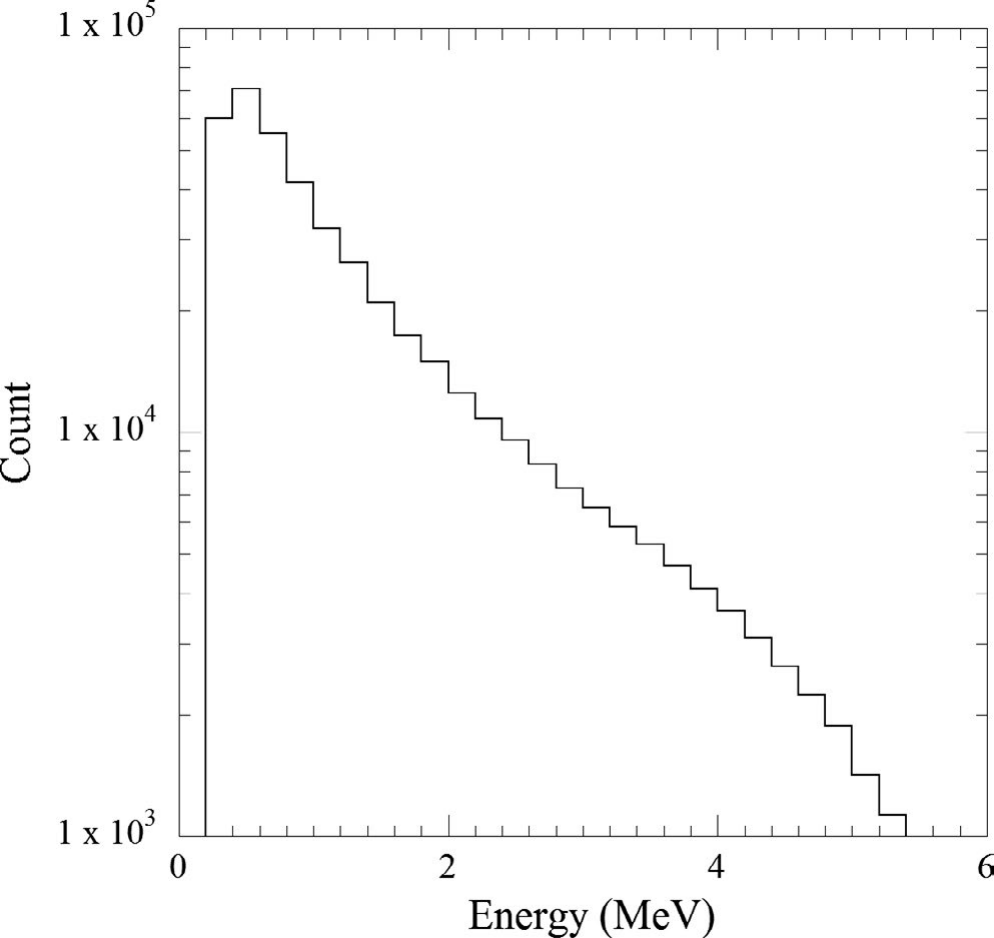
Measured energy spectrum without unfolding

**FIGURE 6:**
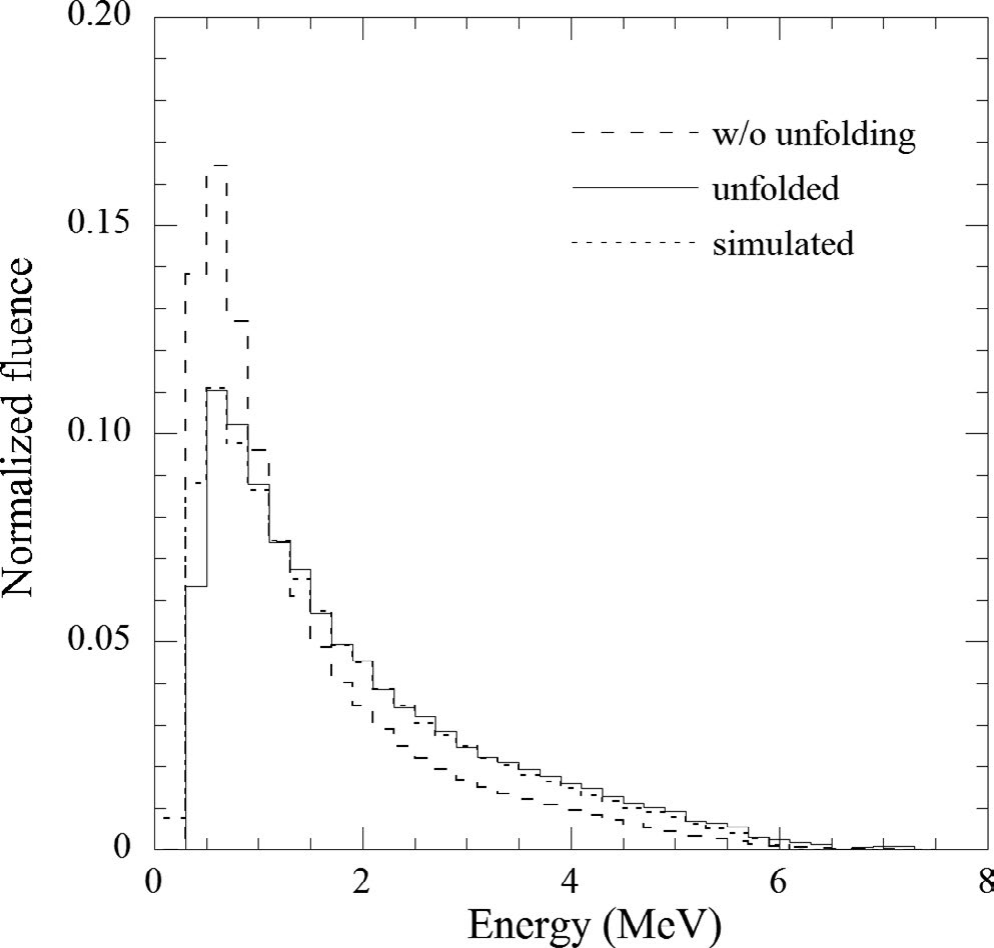
Comparison of normalized energy spectra among measured without unfolding, unfolded after 2000 iterations, and simulated

Figure 5 exhibits the measured count without unfolding. The total measurement count was 4.3 × 10^5^ and the standard deviation was calculated from the three measurement counts was <3.0% up to 5.7 MeV. The normalized energy spectra without unfolding, unfolded after 2000 iterations, and simulated are compared in Figure 6. Moreover, the energy spectrum peak was observed at 0.5 MeV for each energy spectrum, but the mean energies were 1.4, 1.8, and 1.7 MeV for the measured without unfolding, unfolded, and simulated energy spectra, respectively. Several statistical tests were performed to evaluate the differences between those energy spectra. Nonparametric, Wilcoxon's rank‐sum (Wilcoxon), chi‐square (*χ* ^2^), and two‐sample Kolmogorov–Smirnov (two‐sample K‐S) tests were performed because the energy spectra exhibited nonnormal distribution. Table 3 shows the results of the statistical tests with a significance level of 0.05 between the unfolded and simulated energy spectra. No significant difference between the unfolded and simulated energy spectra was observed.

**TABLE 3:** Results of statistical tests between simulated and unfolded energy spectra after 2000 iterations

	Wilcoxon	*χ* ^2^	Two‐sample K‐S
(significance level = 0.05)			
*P*‐value	0.92	1.00	1.00
significant difference	No	No	No

### 3.4 PDD comparison

**FIGURE 7:**
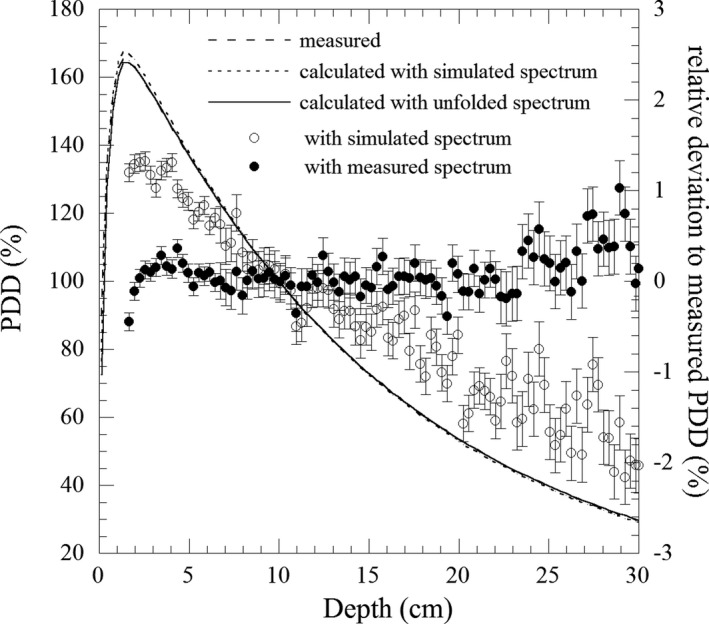
Comparison among measured PDD, calculated PDD using simulated, and unfolded energy spectrum. Plots show relative deviations from measured PDD and error bars are inherent uncertainty of the dose calculation in DOSXYZnrc

Figure 7 shows a comparison of PDD between measurement and calculations, and the calculated PDDs are based on an unfolded energy spectrum and a simulated energy spectrum. The maximum relative deviation between PDD by TM31016 and PDD by our measured energy spectrum was within 1%, whereas it was larger than 2% between the PDD by TM31016 and PDD by the simulated energy spectrum. The rRMSD to TM31016 was 0.25% and 1.07% for PDD based on the measured energy spectrum and the simulated energy spectrum, respectively.

## 4 DISCUSSION

An ultralow dose rate was achieved by adjusting the gun grid voltage in this report. The energy constancy at an ultralow dose rate must be guaranteed. It was confirmed by *TPR*_20,10_ using an ionization chamber and a water tank in this study. A factor of the energy constancy is the 270° bending magnet system. The grid voltage control board was changed to a special pre‐adjusted one to achieve an ultralow dose rate, and it was changed to a normal one by a service engineer after the experiment. Therefore, a disadvantage of the low‐dose‐rate method is that at least 1 day of linac operating time is required for this experiment.

The response function matrix of the NaI(Tl) detector is crucial for the unfolding by the Gold algorithm. We calculated total efficiency *ε*_tot_ and *ε*_p_ of a 7.6 cmϕ × 7.6 cm thickness NaI(Tl) scintillator for a 7.6 cmϕ photon broad beam with EGSnrc and compared with Rogers et al.^30^ to validate the calculated response function. The other calculation parameter was equivalent to the response function computation, and *ε*_tot_ was calculated as the number of counts in all channels relative to the number of incident photons. Moreover, *ε*_tot_ and *ε*_p_ for 0.5 cmϕ were also calculated. Table 4 shows the comparison of *ε*_tot_ and *ε*_p_ between this study and Rogers et al.^30^ for 1, 6, and 20 MeV. Response functions used in this study for unfolding are reliable because both *ε*_tot_ and *ε*_p_ derived in this study showed a good agreement with that by Rogers et al.^30^ In this study, the response function for a 0.5 cmϕ beam was used to unfold the measured spectrum because the photon beam from the linac was collimated with a 0.5 cmϕ hole to shield scattered photons. For a 0.5 cmϕ beam, *ε*_p_ is higher than that in the 7.6 cmϕ broad beam because the escape of the Compton scattered photons decreases. Therefore, when measuring high‐energy X‐rays such as in a linac, the beam width can be reduced to increase the efficiency.

**TABLE 4:** Comparison of peak *ε*° and total efficiency *ε*_tot_ values

Energy of photon (MeV)	Rogers et al.^30^		This study			
	7.6 cmϕ beam		7.6 cmϕ beam		0.5 cmϕ beam	
	*ε*_p_	*ε*_tot_	*ε*_p_	*ε*_tot_	*ε*_p_	*ε*_tot_
1	0.47	0.80	0.47	0.80	0.52	0.80
6	0.12	0.62	0.12	0.62	0.16	0.62
20	0.01	0.70	0.01	0.71	0.02	0.71

For the Gold algorithm, 2000 iterations were recommended by the theoretical simulation using the measured Gaussian distribution of the peak owing to annihilation. In this study, direct energy spectrum measurement at an ultralow dose rate unfolding by 2000 iterations was validated by comparing with a simulated energy spectrum using several statistical. Furthermore, PDDs based on both energy spectra were compared to demonstrate the influence from the energy spectrum obtained in different methods. Statistical tests to estimate the difference in energy spectrum were performed by changing the number of iterations *i* to obtain the optimal number of iterations for the deconvolution of bremsstrahlung. Table 5 shows the *p*‐values of the statistical tests between the unfolded energy spectrum after *i* and that after 2000 iterations and rRMSD between calculated PDDs using the unfolded energy spectrum after *i* and that after 2000 iterations. The *P*‐value of the Wilcoxon rank‐sum test approached one after several iterations, and there was no apparent change in the *p*‐value of the chi‐square and two‐sample Kolmogorov–Smirnov tests. In each test, a significant difference was not found between the absence and presence of the unfolded energy spectrum. The rRMSD of PDD decreased steeply after one iteration. A noticeable change was not found in the number of iteration *i*. Unlike finding an FWHM of photopeak, numerous iterations may not be required for the deconvolution of bremsstrahlung.

**TABLE 5:** *p*‐value of statistical tests between the unfolded energy spectrum after *i* and that after 2000 iterations and rRMSD between calculated PDDs using the energy spectrum after *i* and 2000 iterations

*i*: number of iterations	*p*‐value			PDD rRMSD (%**)**
	Wilcoxon	*χ* ^2^	Two‐sample K‐S	
0	0.32	1.00	0.80	5.31
1	0.94	1.00	1.00	0.54
2	0.95	1.00	1.00	0.53
5	0.99	1.00	1.00	0.31
10	1.00	1.00	1.00	0.31
100	1.00	1.00	1.00	0.29

From the comparison of the PDDs, PDD calculated from unfolded energy spectrum was more agreed with measured PDD than PDD calculated from the simulated energy spectrum. When simulating the energy spectrum, it is necessary to adjust the energy distribution of the incident electrons even if the linac head is faithfully reproduced. The reason for the PDDs difference between the PDD calculated from the simulated energy spectrum and the measured PDD is thought to be insufficient matching of the energy spectrum in MC simulation. Adjustment of the energy spectrum requires the measurement of PDDs in various irradiation fields and sensitive matching of the energy spectrum. To adjust the simulated energy spectrum for each device, it takes a considerable amount of time to measure the PDD and calculate the energy spectrum. Therefore, it is desirable to obtain the energy spectrum by direct measurement.

## 5 CONCLUSION

The X‐ray energy spectrum from a clinical linac was directly measured using a NaI(Tl) scintillator at an ultralow dose rate achieved by adjusting the gun grid voltage. The measured energy spectrum was unfolded by the Gold algorithm and compared with the simulated energy spectrum using statistical tests. Furthermore, the PDD calculated using the unfolded energy spectrum was compared with the measured PDD to evaluate the validity of the unfolded energy spectrum.

Although there was no significant difference between the unfolded and simulated energy spectra by some nonparametric tests, PDD calculated using the unfolded energy spectrum shows better agreement with the measured PDD than the PDD calculated using the simulated energy spectrum. From these results, it was concluded that the feasibility of the direct measurement of the realistic X‐ray energy spectrum from a clinical linac was demonstrated.

## CONFLICT OF INTEREST

No conflicts of interest.

## AUTHOR CONTRIBUTIONS

Y.S. and H.S. conceived of the presented idea. All authors conceived and planned the work that led to the paper. Y.S., M.H., R.Y., and R.M. performed the experiment and A.M., W.C., and H.S. supervised the research. Y.S. analyzed the data and all authors discussed the analysis method and results. All authors wrote the paper, or reviewed successive versions and took part in the revision and approved the final version.
